# Determining of JK*A and JK*B Allele Frequency Distribution among Muslim Blood Donors from Southern Thailand

**DOI:** 10.21315/mjms2019.26.1.5

**Published:** 2019-02-28

**Authors:** Ubonwan Puobon, Kamphon Intharanut, Supattra Mitundee, Oytip Nathalang

**Affiliations:** 1 Graduate Program in Medical Technology, Faculty of Allied Health Sciences, Thammasat University, Pathumtani, Thailand; 2 Graduate Program in Biomedical Sciences, Faculty of Allied Health Sciences, Thammasat University, Pathumtani, Thailand; 3 Regional Blood Centre 12th Songkhla, Thai Red Cross Society, Songkhla, Thailand

**Keywords:** Kidd genotyping, Kidd allele frequencies, southern Thai-Muslims

## Abstract

**Background:**

The Kidd (JK) blood group system is of clinical importance in transfusion medicine. JK*A and JK*B allele detections are useful in genetic anthropological studies. This study aimed to determine the frequencies of JK*A and JK*B alleles among Muslim blood donors from Southern Thailand and to compare how they differ from those of other populations that have been recently studied.

**Methods:**

A cross-sectional study was used. Totally, 427 samples of dissimilar Thai-Muslim healthy blood donors living in three southern border provinces were selected via simple random sampling (aged 17–65 years old) and donors found to be positive for infectious markers were excluded. All samples were analysed for JK*A and JK*B alleles using PCR-SSP. The Pearson’s chi-squared and Fisher exact tests were used to compare the JK frequencies among southern Thai-Muslim with those among other populations previously reported.

**Results:**

A total of 427 donors—315 males and 112 females, with a median age of 29 years (interquartile range: 18 years)—were analysed. A JK*A/JK*B genotype was the most common, and the JK*A and JK*B allele frequencies among the southern Thai-Muslims were 55.2% and 44.8%, respectively. Their frequencies significantly differed from those of the central Thai, Korean, Japanese, Brazilian–Japanese, Chinese, Filipino, Africans and American Natives populations (*P* < 0.05). Predicted JK phenotypes were compared with different groups of Malaysians. The Jk(a+b+) phenotype frequency among southern Thai-Muslims was significantly higher than that of Malaysian Malays and Indians (*P* < 0.05).

**Conclusions:**

The JK*A and JK*B allele frequencies in a southern Thai-Muslim population were determined, which can be applied not only to solve problems in transfusion medicine but also to provide tools for genetic anthropology and population studies.

## Background

The Kidd (JK) blood group system is known to have clinical importance in transfusion medicine. The three antigens—Jk^a^, Jk^b^ and Jk3—are divided into four phenotypes. The Jk(a+b+), Jk(a+b−) and Jk(a−b+) phenotypes are common, in contrast to the Jk(a−b−) phenotype, which is found in less than 0.01% of most populations (1–4) but in 0.1%–1.4% of Polynesians and Finns (5). The JK antibodies, produced after previous transfusions or pregnancies, tend to cause mild delayed haemolytic transfusion reactions (HTRs) and haemolytic disease of the foetus and newborn (HDFN) (1–3).

The Jk^a^ and Jk^b^ antigens are produced by the JK*A and JK*B alleles of a *JK* (*SLC14A1*) gene located on chromosome 18. JK*A/JK*B polymorphism results from a single nucleotide polymorphism (SNP). c.838G>A in exon 9 is associated with an p.Asn280Asp substitution in the JK glycoprotein and red cell urea transporter (1–3, 6). Occasionally, homozygous and compound heterozygous states of inactivating mutations in the *JK* gene, despite encoding JK*A and/or JK*B backgrounds, have led to the JK-null phenotype (5). A urea lysis test is commonly used to identify the Jk(a−b−) phenotype (7, 8). Various molecular techniques for *JK* allele detections that can predict the three common JK phenotypes are polymerase chain reaction (PCR)-based techniques, real-time PCR and microarray-based systems (9–11). However, the PCR-based techniques are appropriate for *JK* allele detections in limited-resource countries. In addition, *JK* allele detections are helpful to avoid certain limitations of serological tests, provide compatible blood unit(s) for patients and enable research in the field of genetic anthropology (12).

*JK* allele frequency distributions may be affected by racial and ethnic differences, migration, disease and mixed marriage. In Thailand, distinct Thai-speaking groups can be categorised as Siamese (Central Thai), North-Eastern Thai (Isan), Northern Thai (Khon Muang), Southern Thai, Thai-Muslims and others (13). The populations of the three southern provinces in Thailand—Pattani, Yala and Narathiwat—are almost entirely Muslim. A recent Diego allele frequency study among the southern Thais revealed that the frequencies significantly differed between the central and northern Thais (14), but the *JK* allele frequencies among the southern Thai-Muslims remain unknown.

This study aimed to determine the frequencies of JK*A and JK*B alleles among Muslim blood donors from Southern Thailand in comparison to those of other populations that have been recently studied.

## Materials and Methods

### Donor Subjects and DNA Preparations

This was a cross-sectional study. Ethylenediaminetetraacetic acid (EDTA)-anticoagulated donated blood samples from dissimilar Thai-Muslims living in the three southern border provinces of Pattani, Yala and Narathiwat were selected via simple random sampling from the Regional Blood Centre 12th Songkhla, Thai Red Cross Society (TRCS) in Songkhla, Thailand. The sample size calculation based on a single proportion formula, this study was based on the largest Jk(a+b+) phenotype prevalence in Thais of 45.3% (9), with a confidence interval of 95% and a margin of error of 4.72%. The calculated sample size of 427 blood donors was sufficient to meet the study objective. Unrelated healthy blood donors aged 17–65 years old were included. The criteria excluded donors with positive infectious marker screenings according to a standard guideline (1). A total of 427 samples were collected from September to October of 2016. All participating volunteers provided their consent after being informed of the study protocols. The Committee on Human Rights Related to Research Involving Human Subjects at Thammasat University in Pathumtani, Thailand approved the study (COE No. 080/2560).

From peripheral blood samples, we extracted genomic DNA using a genomic DNA extraction kit (REAL Genomics, RBCBioscience, Taipei, Taiwan), which was then kept at −20 °C until it was genotyped.

### DNA Controls

Ten identified samples of DNA consisting of 3 Jk(a+b−), 3 Jk(a−b+), 3 Jk(a+b+) and 1 Jk(a− b−) of JK*02N.01 (c.342-1g>a) phenotypes, confirmed by DNA sequencing were used as controls.

### Screening of Jk(a−b−) Phenotype via a Urea Lysis Test

Screening for the Jk(a−b−) phenotype via a direct urea lysis test was performed in all blood samples, as previously described (8). Twenty-five microlitres of 1% red cell suspension in phosphate-buffered saline (PBS) (pH 7.2) were placed in each well of a microplate. Thereafter, 50 μL of 2M urea diluted in distilled water was added, mixed and incubated at room temperature for 5 min and then centrifuged at 1,800 rpm for 2 min (Universal 320/320R centrifuge, Hettich Lab Technology, Tuttlingen, Germany). The plate was read for haemolysis by the naked eye. A Jk(a+b+), negative control (O_1_ or O_2_ screening cells, National Blood Centre, TRCS, Bangkok, Thailand) and a Jk(a−b−), positive control for haemolysis were included. Complete haemolysis within 5 min of incubation demonstrated a negative reaction for the phenotypes of Jk(a+b−), Jk(a−b+) and Jk(a+b+). A non-haemolytic reaction within 5 min of incubation could be found only in the Jk(a−b−) phenotype.

### Detection of JK*A and JK*B Alleles Using PCR-SSP

Detection of JK*A and JK*B alleles was carried out using standard PCRSSP, as previously described, with some modifications (15). In brief, 1 μL of genomic DNA (50 ng/μL) was amplified in 10 μL of total volume (1 μL of 5 μM JK-AB-Forward primer 5′-CATGCTGCCATAGGATCATTGC-3′ and 1 μL of 5 μM JK-A-Reverse primer 5′-CCAGAGTCCAAAGTAGATGTC-3′) to detect the JK*A allele. For JK*B allele detection, 1 μL of 5 μM JK-AB-Forward primer and 1 μL of 5 μM JK-A-Reverse primer 5′-CCAGAGTCCAAAGTAGATGTT-3′ were used. The human growth hormone (HGH) gene was co-amplified with 1 μL of 3 μM HGH-Forward primer 5′-TGCCTTCCCAACCATTCCCTTA-3′, and 1 μL of 3 μM HGH-Reverse primer 5′-CCACTCACGGATTTCTGTTGTGTTTC-3′ was used as an internal control. A standard PCR technique was used with the reaction mixture of 5 μL of 2X PCR (OnePCR Plus, GeneDirex, Taiwan) using a T100 Thermal Cycler (Bio-Rad Laboratories, Inc., Hercules, CA, USA).

The PCR technique consisted of one cycle of 95 °C for 5 min, followed by 30 cycles at 95 °C for 30 s, 61 °C for 40 s and 72 °C for 30 s. The final step was a 5-min extension at 72 °C, followed by storage at 10 °C. After amplifying, the newly created products were electrophoresed at 100 volts with a 1.5% agarose gel using 1X Trisborate-EDTA (TBE) buffer containing a 10,000× fluorescent DNA gel stain (SYBR Safe DNA gel stain, Invitrogen, Paisley, UK) and visualised using blue-light illumination. The product size of the PCR samples for both JK*A and JK*B alleles was 301 bp, whereas that of the *HGH* gene internal control was 434 bp.

### DNA Sequencing

The results of the PCR-SSP were confirmed by sequencing the genomic DNA of 20 genotype donors (five JK*A/JK*A, 10 JK*A/JK*B and five JK*B/JK*B). After amplifying the genomic DNA, a 430 bp fragment that contained SNPs (c. 838G/A) was obtained using the JK-AB-Forward primer and reverse primer 5′-TAGTCATGAGCAGCCCTCCCC-3′. Similarly, the PCR technique was used for JK*A and JK*B genotyping.

### Statistical Analysis

Gene and allele frequencies among southern Thai-Muslims were estimated by gene counting. The agreement between the observed and expected values of genotype frequencies was tested using the Hardy-Weinberg equilibrium and a chi-squared (*χ*^2^) test (16). A Pearson’s chi-squared test was conducted between the independent variables of Kidd allele frequencies in southern Thai-Muslims and the independent variables of previously reported populations (11, 17–25) using the allele frequencies in a 2 × 2 contingency table to determine whether the allele frequencies of southern Thai-Muslims significantly differed from those of other population. In addition, Pearson’s chi-squared and Fisher’s exact tests were used to test possible associations using a 2 × 2 contingency table to demonstrate any differences among independent variables in the frequencies of Kidd predicted phenotypes between the southern Thai-Muslim and Malaysian populations (26). All statistical analyses were conducted using SPSS, Version 16.0 (SPSS Inc., Chicago, IL, USA). A *P*-value less than 0.05 was established as significant.

## Results

A total of 427 donors—315 males and 112 females with a median age of 29 years (interquartile range: 18 years)—were analysed. To screen for the Jk(a−b−) phenotype, all 427 samples produced negative results using the urea lysis test. The results of a two-tube PCR-SSP were used to distinguish between JK*A and JK*B alleles. The first and second mixes could differentiate between JK*A and JK*B alleles with an amplified product size of 301 bp, similar to the results of a related study (15). The validated genotyping results of 10 DNA controls were consistent with each other, and 20 DNA samples tested by PCR-SSP showed 100% concordance with the DNA sequencing results.

### JK*A and JK*B Frequencies among Southern Thai-Muslims

The JK*A and JK*B genotype and allele frequencies among southern Thai-Muslims are shown in Table 1. A total of 427 DNA samples from southern Thai-Muslims were examined for the JK*A and JK*B alleles using the standard PCR-SSP technique. JK*A/JK*B was the most common genotype (229/427), followed by JK*A/JK*A (121/427) and JK*B/JK*B (77/427). The JK genotypes of the 427 southern Thai-Muslims determined in this study were consistent with each other according to the Hardy-Weinberg equilibrium (*χ*^2^ = 3.101, DF = 1, *P* = 0.078). The JK*A and JK*B allele frequencies among the Southern Thai-Muslims were 55.2% (471/854) and 44.8% (383/854), respectively.

### Comparison of JK*A and JK*B Allele Frequencies Across Populations

The frequencies of JK*A and JK*B alleles were compared among Thais and other ethnic groups (Table 2). The observed allele frequencies of the southern Thai-Muslims were similar to those found in northern Thai, Han Chinese, South Asian, Southeast Asian, Hispanic, Alaskan Native, Pacific Islander, southern Brazilian and Caucasian populations. On the contrary, the allele frequencies of southern Thai-Muslims significantly differed (*P* < 0.05) from those of central Thai, Korean, Japanese, Brazilian–Japanese, Chinese, Filipino, African and American Native populations.

### Comparison of JK Phenotypes among Southern Thai-Muslims and Malaysians

The JK genotyping results of southern Thai-Muslims were computed to three predicted phenotypes—Jk(a+b−), Jk(a−b+) and Jk(a+b+)—and compared among different groups of Malaysian populations (Table 3). The Jk(a+b+) phenotype was the most common among southern Thai-Muslims and Malaysians, but its frequency among southern Thai-Muslims was significantly higher than among Malaysian Malays (53.6% versus 43.0%, *P* = 0.013) and Malaysian Indians (53.6% versus 43.3%, *P* = 0.046). Moreover, the frequency of the Jk(a−b+) phenotype among southern Thai-Muslims was significantly lower than that among Malaysian Chinese (18.0% versus 24.8%, *P* = 0.031). A rare Jk(a−b−) phenotype was found only in Malaysian Malays and Malaysian Indians.

## Discussion

In this study, JK*A and JK*B alleles were detected in 427 Muslim blood donors from Southern Thailand with in-house PCR-SSP. The genotyping results computed to three predicted phenotypes with the exclusion of the Jk(a−b−) phenotype because all samples were negative, as revealed by the urea lysis test. The validated in-house PCR-SSP genotyping results were in accordance with the DNA sequencing results; hence, the JK typing results were accurate and reliable.

Thereafter, the JK*A and JK*B genotypes and allele frequencies were calculated. It was demonstrated that the most common was the heterozygous JK*A/JK*B, followed by the JK*A/JK*A and JK*B/JK*B genotypes. The predicted phenotypes of Jk(a+b−), Jk(a−b+) and Jk(a+b+) were computed and compared across populations. A high prevalence of the Jk(a+b−) phenotype among southern Thai-Muslims may have resulted in an increased possibility of anti-Jk^b^ alloimmunisation among patients after blood transfusions, which was similar to Malaysian Malays and Malaysian Indians (26). In contrast, a related report regarding central and northern Thais revealed that the percentages of Jk(a+b−) and Jk(a−b+) phenotypes were nearly the same, leading to an equal ratio of anti-Jk^a^ and anti-Jk^b^ alloimmunisations (17).

Concerning population genetics, JK*A and JK*B alleles could be used as tools to study the relationships among populations. The allele frequencies among Muslims from Southern Thailand were related to those of northern Thais, south and southeast Asians, similar to a related DI*A and DI*B allele frequency study in three populations in Thailand (14). This may be because the populations are in the same geographic region. Similarly, American Natives and Africans were in an area to the far west, resulting in significantly differing JK*A and JK*B frequencies from those of southern Thai-Muslims. In addition to geographic region, other factors come into play (e.g., homogeneous populations may be involved in the differing of allele frequencies between Thai-Muslims and eastern Asians, including Japanese, Korean and Chinese) (27).

The people of the three southern provinces of Thailand live along the Thai-Malaysian border and share strong ethnic, linguistic, religious and cultural bonds with the people across the border. In addition to these factors involved in the relationships of southern Thai-Muslims and Malaysians, genetic similarities may be further evidence of either isolation or interaction among these populations (28). In our study, the Jk(a+b+) phenotype frequency among southern Thai-Muslims was significantly higher than those of both Malaysian Malays and Indians. However, a similar pattern of JK phenotypes—Jk(a+b+) > Jk(a+b−) > Jk(a−b+) phenotypes—was observed and was consistent with that of ethnic groups (Malay) in neighbouring southern Thailand (26). Meanwhile, the Jk(a−b+) phenotype frequency among southern Thai-Muslims was significantly lower than that among Malaysian Chinese, whose patterns were similar to those of central and northern Thais, likely due to mixing with Chinese lineages (17). Additional studies of further appropriate blood group alleles using more samples are required to authenticate these findings.

## Conclusion

The frequencies of JK*A and JK*B alleles in a population of Muslim blood donors from Southern Thailand were determined. This data can be applied not only to reduce problems in transfusion medicine but also to provide a tool for genetic anthropology and population studies.

## Figures and Tables

**Table 1:** JK*A and JK*B genotype and allele frequencies among southern Thai Muslims

(427 donors x 2 alleles)		Genotype	Observed (%)	Expected (HWE)	*χ*^2^	*P*-value

Allele	Allele frequency (%)					
JK*A	471 (55.2)	JK*A/JK*A	121 (28.4)	130		
JK*B	383 (44.8)	JK*A/JK*B	229 (53.6)	211	3.101	0.0783
		JK*B/JK*B	77 (18.0)	86		

**Table 2:** JK*A and JK*B allele frequencies among populations

Populations	Number	Allele frequency (%)		Methods	Pearson’s *χ*^2^ test between Southern Thai Muslim and other populations	
	
		JK*A	JK*B		*χ*^2^	*P*-value
Thais						
Southern Thai Muslim	427	471 (55.2)	383 (44.8)	PCR-SSP	-	-
Central Thai (16)	500	**503 (50.3)**	**497 (49.7)**	PCR-SSP	4.157	0.042
Northern Thai (16)	300	299 (49.8)	301 (50.2)	PCR-SSP	3.791	0.052
Asians						
Korean (11)	1,033	**988 (47.8)**	**1,078 (52.2)**	Microarray	12.696	< 0.001
Japanese (11)	1,022	**987 (48.3)**	**1,057 (51.7)**	Microarray	11.081	< 0.001
Brazilian-Japanese (17)	209	**193 (46.2)**	**225 (53.8)**	PCR-RFLP	8.714	0.003
Chinese (11)	1,715	**1,590 (46.4)**	**1,840 (53.6)**	Microarray	20.843	< 0.001
Chinese (Shanghai) (18)	403	**382 (47.4)**	**424 (52.6)**	Microarray	9.681	0.002
Filipino (11)	1,333	**1,302 (48.9)**	**1,364 (51.1)**	Microarray	10.067	0.002
Han Chinese (Jiangsu) (19)	146	148 (50.6)	144 (49.3)	PCR-SSP	1.573	0.210
South Asian (11)	922	1,056 (57.3)	788 (42.7)	Microarray	0.978	0.323
Southeast Asian (11)	942	991 (52.6)	893 (47.4)	Microarray	1.436	0.231
Africans						
African American (20)	690	**1,001 (72.5)**	**379 (27.5)**	Microarray	70.163	< 0.001
Mali (21)	300	**461 (76.8)**	**139 (23.2)**	Luminex	71.047	< 0.001
Americans						
American Native (11)	970	**977 (50.4)**	**963 (49.6)**	Microarray	5.262	0.022
Hispanic (20)	119	136 (57.1)	102 (42.9)	Microarray	0.224	0.636
Alaska Native/Aleut (11)	621	649 (52.3)	593 (47.7)	Microarray	1.593	0.207
Hawaiian/Pacific Islander (11)	522	590 (56.6)	454 (43.4)	Microarray	0.300	0.584
Southern Brazilians						
Santa Catarina (22)	373	396 (53.1)	350 (46.9)	PCR-RFLP	0.606	0.436
Paraná (23)	400	410 (51.3)	390 (48.7)	PCR-RFLP	2.372	0.124
Caucasians						
Caucasian (20)	1,243	1,293 (52.0)	1,193 (48.0)	Microarray	2.392	0.122
French Basque (24)	114	129 (56.6)	99 (43.4)	PCR-ASP	0.096	0.756

PCR-SSP: PCR with sequence specific primers; PCR-RFLP: PCR with restriction fragment length polymorphism; PCR-ASP: PCR with allele-specific primers.

In bold, frequencies differed from those among southern Thai Muslims (*P* < 0.05).

**Table 3:** Frequencies of JK predicted phenotypes among Southern Thai Muslims and Malaysians

Phenotype	Phenotypes frequency (%)				*P*-value

	Southern Thai Muslim	Malaysian Malay	Malaysian Indian	Malaysian Chinese	
Southern Thai Muslim versus Malaysian Malay					
Jk(a+b−)	121 (28.4)	72 (36.0)			0.052
Jk(a−b+)	77 (18.0)	35 (17.5)			0.863
Jk(a+b+)	229 (53.6)	86 (43.0)			0.013
Jk(a−b−)	0 (0.0)	7 (3.5)			**0.000**^1^
Southern Thai Muslim versus Malaysian Indian					
Jk(a+b−)	121 (28.4)		42 (35.0)		0.158
Jk(a−b+)	77 (18.0)		24 (20.0)		0.624
Jk(a+b+)	229 (53.6)		52 (43.3)		0.046
Jk(a−b−)	0 (0.0)		2 (1.7)		**0.048**^1^
Southern Thai Muslim versus Malaysian Chinese					
Jk(a+b−)	121 (28.4)			67 (24.5)	0.258
Jk(a−b+)	77 (18.0)			68 (24.8)	0.031
Jk(a+b+)	229 (53.6)			139 (50.7)	0.454
Jk(a−b−)	0 (0.0)			0 (0.0)	NA

NA: not applicable

1 Fisher’s exact test

The combination of other phenotypes was used as the reference group to compare with an interested predicted phenotype. In bold, frequencies differed from those among southern Thai Muslims (*P* < 0.05).
